# Large language models for breast cancer treatment planning: a blinded real-world evaluation of DeepSeek, ChatGPT, and oncologist recommendations

**DOI:** 10.3389/fdgth.2026.1862730

**Published:** 2026-06-30

**Authors:** Ming Li, Yiran Yu, Gang Li, Xiaoli Zhang, Yuting Shi, Rila Su

**Affiliations:** 1 Department of Health Policy Management, Bloomberg School of Public Health, Johns Hopkins University, Baltimore, MD, United States; 2 Graduate School of Inner Mongolia Medical University, Hohhot, China; 3 Oncology Department of Inner Mongolia People's Hospital, Hohhot, China; 4 Clinical Medical Research Center of Inner Mongolia People's Hospital, Hohhot, China

**Keywords:** accuracy, artificial intelligence, breast cancer, large language models, variability analysis

## Abstract

**Rationale and objectives:**

Large language model (LLM) are increasingly explored for oncology decision support, yet their alignment with real-world clinical practice across varying disease complexities remains insufficiently characterized. This study aimed to evaluate and compare the accuracy, stability, and concordance of two advanced LLMs—DeepSeek V3.1 and ChatGPT-5—against experienced oncologists in generating breast cancer treatment plans within a specific clinical setting.

**Materials and methods:**

This retrospective study compared the performance of DeepSeek V3.1 and ChatGPT-5 with senior oncologists using de-identified records from 213 breast cancer patients (Stages I–IV). To assess effectiveness, we implemented a multidimensional evaluation framework: accuracy was measured using a 5-point Likert scale by three independent, blinded expert reviewers; internal consistency was quantified via variance and coefficient of variation; and clinical concordance was evaluated using a structured five-level scoring system. Statistical analyses, including ANOVA and ordinal regression, were used to examine the impact of disease stage on AI-human agreement.

**Results:**

Under standardized retrospective review conditions, LLM-generated recommendations demonstrated higher expert-rated guideline concordance and lower variability than historical real-world oncologist plans. Specifically, DeepSeek V3.1 achieved the highest expert-rated accuracy scores with minimal internal variance (4.91 ± 0.36), outperforming both ChatGPT-5 (4.65 ± 0.62) and clinicians (3.82 ± 0.63, *P* < 0.001). While AI outputs exhibited high mutual consistency (74.2%), expert evaluations revealed a significant decline in AI-clinician agreement as disease stage advanced (*P* < 0.001), particularly in Stage IV cases where clinicians prioritized real-world constraints such as financial toxicity.

**Conclusions:**

Advanced LLMs, particularly DeepSeek V3.1, demonstrated strong performance in generating standardized, guideline-concordant breast cancer treatment plans, showing superior consistency over human specialists in protocol-driven scenarios. However, the widening gap in complex late-stage cases highlights limitations in accounting for clinical context and socioeconomic factors. These findings support the role of LLMs as robust clinician-supervised decision-support tools while emphasizing the necessity of human judgment for individualized care.

## 1 Introduction

Breast cancer is the most common malignancy in women globally and in China. In 2022, there were ∼2.3 million new cases and 670,000 deaths worldwide, with ∼357,200 new cases in China (1, 2). Rising incidence highlights the urgent need for standardized, individualized treatment.

Large language models (LLMs) have been studied and applied in the medical field, The integration of Artificial Intelligence, particularly Large Language Models (LLMs), is rapidly transforming healthcare demonstrating significant potential in areas such as interpretation of radiology diagnostic reports, generation of clinical summaries, and assistance in diagnostic and treatment decision-making (3, 4). Many studies have explored the application of LLMs in breast cancer care, particularly in text generation and providing guideline-based responses (5, 6). These efforts highlight the potential of LLMs as decision-support tools in multidisciplinary breast tumor board settings (7).

With the growing implementation of these tools, selecting an appropriate model has become crucial. ChatGPT widely recognized as a global benchmark in the LLM landscape, has demonstrated strong capabilities in general medical reasoning (8, 9). At the same time, domestic models such as DeepSeek are gaining significant traction in China (10). DeepSeek has been progressively piloted in clinical care, hospital management, and research across numerous Chinese hospitals. While ChatGPT represents the current international state-of-the-art, DeepSeek offers distinct advantages in processing Chinese medical terminology and aligning with local clinical guidelines.

Currently, research on the applicability of LLM in real-world clinical decision-making for breast cancer in China remains limited, particularly lacking high-quality validation studies that use complete, real patient records as input and conduct blinded comparisons with actual clinical treatment plans. Performance in vertical medical scenarios—especially in recommending oncology treatment regimens—requires systematic evaluation.

In oncology clinical decision-making, the management pathway for early-stage breast cancer is relatively well-established, whereas advanced disease requires dynamic trade-offs amidst high heterogeneity, therapeutic uncertainty, and real-world constraints (11). Consequently, traditional evaluation methods relying solely on average accuracy may obscure critical differences across key dimensions such as variability, dispersion, and consistency in different decision-making scenarios.

From a systems perspective, the output of large language models (LLMs) can be viewed as a probabilistic encoding of guideline knowledge distributions (12, 13), while clinical decision-making by physicians represents an optimized outcome integrating complex constraints such as individual patient characteristics, economic factors, and institutional considerations (14). The differences in decision-making architectures between the two may not only manifest in average performance but may also be profoundly reflected in variance structures and patterns of decision consistency related to disease stage.

To address this research gap, this study proposes a variance-based evaluation framework that moves beyond simple accuracy comparisons. It systematically characterizes the differences between treatment recommendations generated by large language models (LLMs) and actual clinical decisions across multiple dimensions, including accuracy, dispersion, and consistency degradation. By designing a blinded review process and robust statistical models, this framework quantifies within-model variation, between-model consistency, and stage-specific differences based on a real-world patient cohort. It shifts the analytical focus from isolated performance comparisons to the structured modeling of decision-making processes, thereby laying a methodological foundation for the evaluation and deployment of LLM systems in oncology.

Within this framework, and in response to the scarcity of high-quality validation studies for clinical decision-making in breast cancer in China—particularly those lacking complete medical records as input and blinded comparisons with actual treatment plans—this study plans to conduct a retrospective, blinded, expert-reviewed investigation. It will systematically compare the accuracy and potential innovativeness of DeepSeek V3.1 and GPT-5 in formulating treatment plans for Chinese breast cancer patients. The aim is to provide support for generating high-level clinical evidence and to validate the feasibility of using large language models to assist oncology experts in personalized decision-making.

## 2 Methods

### 2.1 Study design and conceptual framework

This study employed a retrospective comparative design to evaluate the accuracy and clinical applicability of large language models in generating individualized treatment plans for breast cancer. The research process primarily included five key steps: case selection, integration of medical record data, generation of treatment plans by large language models, blinded expert review, and statistical analysis. Informed consent was obtained from all human participants involved in the research.

This study adopts a retrospective comparative design, aiming to systematically evaluate the structural characteristics of tumor treatment plans generated by large language models (LLMs). Its core conceptual framework is based on a key distinction: viewing the output of LLMs as probabilistic encoding of clinical guideline knowledge, while understanding the plans selected by clinicians as decision-making results that undergo adaptive optimization in specific contexts. Within this framework, the research evaluation is not limited to comparing differences in average performance, but explicitly models the following aspects through a variance-based model: the distribution of decision accuracy, the dispersion of recommendation results, the dynamics of consistency, and the consensus degradation patterns that vary with disease stage.

The study protocol was approved by the Ethics Committee of the Inner Mongolia People's Hospital (Approval No. 202306209L).

### 2.2 Patients cohort

The study consecutively enrolled breast cancer patients diagnosed and receiving systemic treatment at the oncology department and breast surgery department of the hospital starting from January 2022 to October 2024.

#### 2.2.1 Inclusion criteria

The inclusion criteria include patients were required to have a first pathological diagnosis of breast cancer after the specified date and to have initiated systemic anti-tumor therapy. Based on the line of treatment, patients were categorized into treatment-naïve patients (receiving first-line treatment for the first time) and subsequent-line treatment patients (receiving second-line or higher treatment after failure of first-line therapy). To ensure sufficient statistical power for subsequent subgroup analyses, included cases must have complete core clinical information, including clear pathological type, clinical stage, molecular subtype, and well-documented treatment line records.

#### 2.2.2 Exclusion criteria

Exclusion criteria included any of the following: concurrent presence of two or more primary malignant tumors; unclear definition of treatment line; being in the terminal stage of disease at the start of the study (defined as ECOG performance status score ≥3 or clinically estimated survival expectancy less than 3 months); or missing key data that prevents short-term efficacy and safety evaluation.

### 2.3 Data collection and preprocessing

#### 2.3.1 Construction of input for large language models

To ensure the completeness and consistency of input data, this study extracted structured and unstructured information for each patient from the hospital's electronic medical record system. The data for study include but not limited the patient's demographics, menopausal status, ECOG status, comorbidities, organ function, pathology, receptor status, HER2/ER/PR/Ki-67, genomic markers, staging details, metastatic sites, imaging findings, main symptoms, signs and the evolution of the condition recordsprevious treatments and current treatment plan. The imaging and pathology images were excluded. All case information was de-identified and used as unified input material.

#### 2.3.2 LLM treatment plan generation

To minimize prompt bias to the greatest extent, a standardized prompt template was established for all cases: “*You are an oncologist. Based on the patient's information, please provide an appropriate treatment plan for this patient, including precautions. Requirements: Provide specific drug treatment regimens, dosages, and cycles. Surgical plans, radiotherapy plans.”*

Large language model evaluations were conducted between October 10 and 24, 2025, using web-based interfaces. GPT-5 was accessed via the ChatGPT platform (OpenAI), and DeepSeek-V3.1 via the official DeepSeek website. Default platform settings were used for both models. Web browsing, document uploads, custom functions, persistent memory, and additional system instructions were disabled whenever possible. No guideline documents or external materials were uploaded.

All prompts were entered in Chinese using a standardized template with identical structured case summaries provided to both models. To assess response stability and reproducibility, each case was independently re-evaluated after a 7-day interval using the same prompt.

#### 2.3.3 Structured data entry and plan organization

The research team entered three plans for each patient—the oncologist's plan in EMR, the DeepSeek-generated plan, and the ChatGPT-generated plan—into a unified electronic database. The three sets of plans for each patient were arranged in parallel in the database and randomly numbered for subsequent blinded review.

### 2.4 Evaluation methods

Expert Review Team and Blinded Design.

The review team consisted of three oncology experts with over 15 years clinical practice. A fully blinded design was adopted for the review: all plans had source identifiers removed and were assigned random numbers. The three groups of plans for the same patient were presented to the reviewing experts in random order, ensuring that experts conducted independent evaluations without knowing the source of the plans.

#### 2.4.1 Accuracy scoring framework

Experts were required to provide an overall score for each plan based on a five-point Likert scale, with evaluation dimensions including the rationality of the treatment strategy, accuracy of core drug selection, standardization of dosage and cycles, appropriateness of local treatment recommendations, and completeness of supportive care and risk warnings. The scoring criteria were defined as: 5 points (fully consistent with clinical judgment), 4 points (mostly consistent), 3 points (basically feasible), 2 points (with obvious deviations), 1 point (infeasible).

Expert reviewers determined optimal treatment plans by integrating CSCO (2025 edition.), NCCN (v2.2025), ASCO (2025 updates), and ESMO (v1.2, 2025), synthesized with best clinical practice.

If the recommended content of a plan exceeds the scope of currently widely used clinical guidelines but aligns with high-level cutting-edge evidence published in the industry within the past year, it is scored according to the five-point scale.

#### 2.4.2 Concordance grading

Meanwhile, design consistency grading standards to quantify the plans among three groups, categorized as complete consistency (≥95%), mostly consistent (60%–94%), partially consistent (30%–59%), mostly inconsistent (5%–29%), and complete inconsistency (<5%). Consistency codes were defined as:

1. (Complete consistency): Core drugs, dosages, cycles, local treatments, and surgical timing are all identical.
2. (Mostly consistent): Core drug types are consistent, with differences only in dosages, cycles, or details of non-core second-line plans.
3. (Partially consistent): Core treatment strategies are consistent (e.g., both endocrine-based), but there are significant differences in primary drugs or local treatments (such as surgery or radiotherapy).
4. (Mostly inconsistent): Core treatment strategies are completely different (e.g., one chooses endocrine therapy, the other chemotherapy), but pathological or imaging bases are interpretable.
5. (Complete inconsistency): Treatment strategies are completely different, and one plan has clear fundamental errors (e.g., using HER2-targeted drugs for HER2-negative cases).

Dispute Resolution Mechanism.

If the score of the same plan exceeds 2 points between any two experts, then the three experts jointly review the plan and reach a consensus score through thorough discussion.

### 2.5 Statistical analysis methods

#### 2.5.1 Descriptive analysis

Descriptive statistical analysis was performed on the baseline characteristics of all enrolled patients. Categorical variables were expressed as frequencies (percentages), and continuous variables as mean ± standard deviation or median (interquartile range). The expert scoring results for the three types of plans were described using mean, standard deviation, minimum, and maximum values.

#### 2.5.2 Consistency comparison

One-way analysis of variance (ANOVA) was used to compare whether there were statistically significant differences in the average scores among the three groups of plans. Paired samples t-tests were performed on the scores of “DeepSeek plan vs. clinical actual plan” and “ChatGPT plan vs. clinical actual plan,” respectively. The distribution proportions of the two LLM-generated plans and the clinical actual plans across different consistency levels were calculated.

#### 2.5.3 Variability and dispersion analysis

Variability and dispersion in treatment recommendations were quantified at the individual level using standard deviation (SD) and coefficient of variation (CV), while group-level differences in variance were assessed through Levene's test and the Brown–Forsythe robust variance test. To further characterize the structural sources of score variability, variance decomposition analysis was conducted to estimate the relative contributions of model type and disease stage to overall decision dispersion.

## 3 Results

### 3.1 Accuracy evaluation

**Figure 1:**
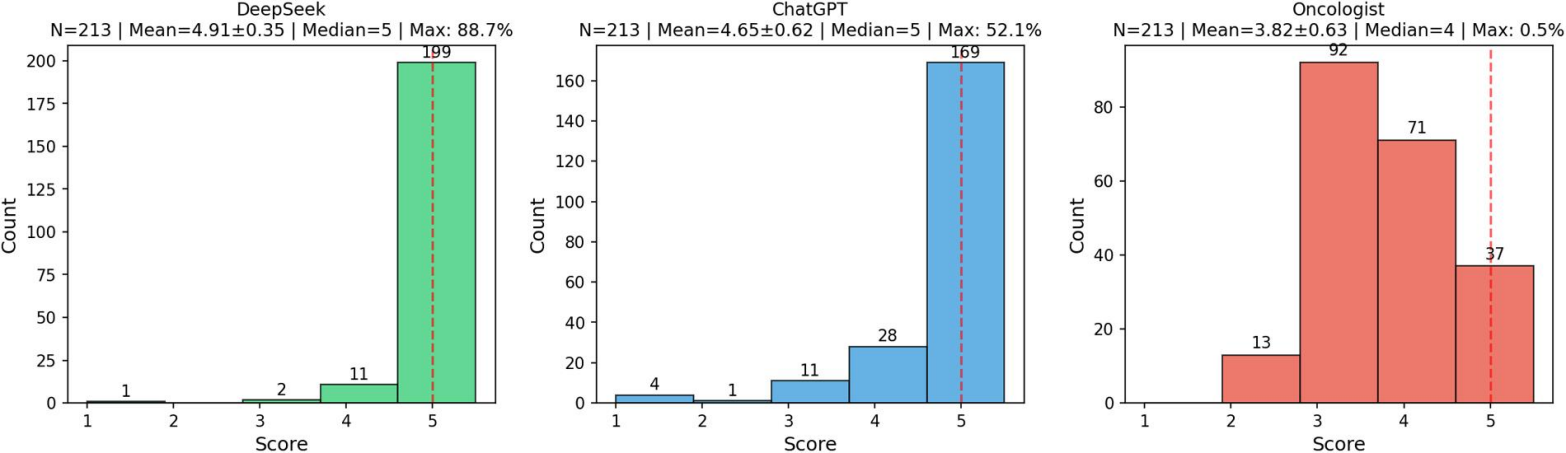
Distribution of evaluation scores.

In this cohort of 213 female patients (mean age 53.07 ± 12.13 years), Significant inter-group differences in treatment recommendation scores were observed. The mean scores for Group DeepSeek, Group ChatGPT, and Group Oncologist were 4.91 ± 0.35, 4.65 ± 0.62, and 3.82 ± 0.63, respectively. Median scores were 5 for both AI groups (DeepSeek and ChatGPT) and 4 for Group Oncologist. Variance analysis showed the highest consistency in Group DeepSeek (0.126), compared to Group ChatGPT (0.381) and Group Oncologist (0.400) (Figure 1).

Paired t-tests indicated that Group DeepSeek significantly outperformed both Group ChatGPT and Group Oncologist (Table 1).

**Table 1:** Pairwise comparisons.

Cohort	Mean Difference	95% CI	t value	*p* value
Oncologist vs. ChatGPT	−0.833	[−0.952, −0.714]	13.752	<0.001
Oncologist vs. DeepSeek	−1.089	[−1.187, −0.991]	21.924	<0.001
ChatGPT vs. DeepSeek	−0.256	[−0.352, −0.160]	5.262	<0.001

### 3.2 Stage-Specific comparisons

Analysis by stage (I: *n* = 24; II: *n* = 75; III: *n* = 70; IV: *n* = 44) yielded:

Stages I–III: A significant hierarchy was observed with Group DeepSeek > Group ChatGPT > Group Oncologist (all *p* < 0.05).
Stage IV: Group DeepSeek ≈ Group ChatGPT > Group Oncologist; Group Oncologist remained significantly inferior to both Group ChatGPT and Group DeepSeek (*p* < 0.001), while no significant difference was found between Group ChatGPT and Group DeepSeek (*p* = 0.74).

### 3.3 Concordance comparison

Across the overall cohort, concordance between the two AI systems (ChatGPT vs. DeepSeek) was substantially higher than that between either AI system and the oncologist. Nearly three quarters of AI–AI comparisons fell into the “≥60% similarity” categories, whereas AI–human comparisons were predominantly distributed in the “30%–59%” and lower similarity ranges.

This pattern was consistent across all disease stages. In Stage I–III, ChatGPT and DeepSeek showed a high proportion of “mostly identical” or “identical” classifications, while agreement with oncologists was markedly lower and shifted toward partial or low similarity. In Stage IV, although AI–AI agreement slightly decreased, it remained clearly higher than AI–human agreement, with the latter showing the largest proportions of low-consistency categories (<30%) (Table 2)

**Table 2:** Concordance comparisons.

Cohort/Stage	Comparison	≥95%	60%–94%	30%–59%	5%–29%	<5%
Overall (*N* = 213)	ChatGPT vs. DeepSeek	18.8% (40)	55.4% (118)	19.7% (42)	5.2% (11)	0.9% (2)
	ChatGPT vs. Oncologist	1.4% (3)	21.6% (46)	37.6% (80)	27.2% (58)	12.2% (26)
	DeepSeek vs. Oncologist	1.4% (3)	22.5% (48)	43.7% (93)	21.1% (45)	11.3% (24)
Stage I (*N* = 24)	ChatGPT vs. DeepSeek	25.0% (6)	66.7% (16)	8.3% (2)	0.0% (0)	0.0% (0)
	ChatGPT vs. Oncologist	0.0% (0)	4.2% (1)	58.3% (14)	25.0% (6)	12.5% (3)
	DeepSeek vs. Oncologist	0.0% (0)	12.5% (3)	54.2% (13)	25.0% (6)	8.3% (2)
Stage II (*N* = 75)	ChatGPT vs. DeepSeek	16.0% (12)	57.3% (43)	20.0% (15)	6.7% (5)	0.0% (0)
	ChatGPT vs. Oncologist	1.3% (1)	21.3% (16)	37.3% (28)	28.0% (21)	12.0% (9)
	DeepSeek vs. Oncologist	1.3% (1)	24.0% (18)	44.0% (33)	17.3% (13)	13.3% (10)
Stage III (*N* = 70)	ChatGPT vs. DeepSeek	21.4% (15)	50.0% (35)	22.9% (16)	4.3% (3)	1.4% (1)
	ChatGPT vs. Oncologist	1.4% (1)	17.1% (12)	31.4% (22)	31.4% (22)	18.6% (13)
	DeepSeek vs. Oncologist	1.4% (1)	20.0% (14)	40.0% (28)	24.3% (17)	14.3% (10)
Stage IV (*N* = 44)	ChatGPT vs. DeepSeek	15.9% (7)	52.3% (23)	27.3% (12)	4.5% (2)	0.0% (0)
	ChatGPT vs. Oncologist	0.0% (0)	27.3% (12)	34.1% (15)	22.7% (10)	15.9% (7)
	DeepSeek vs. Oncologist	0.0% (0)	25.0% (11)	38.6% (17)	22.7% (10)	13.6% (6)

After stratification by clinical stage (Stages I–IV), concordance grades were significantly associated with disease stage for all three comparisons. Kruskal–Wallis tests demonstrated significant between-stage differences (ChatGPT vs. DeepSeek *χ*² = 72.1; ChatGPT vs. Oncologist*χ*² = 60.2; DeepSeek vs. Oncologist *χ*² = 59.5; all *P* < 0.001). Ordinal logistic regression revealed a monotonic decline in agreement with advancing stage. For each one-stage increase, the odds of being in a worse agreement category increased substantially: OR = 28.0 (95% CI: 8.7–90.2; *p* < 0.001) for ChatGPT vs. DeepSeek, OR = 4.7 (95% CI: 2.9–7.5; *p* < 0.001) for ChatGPT vs. Oncologist, and OR = 4.7 (95% CI: 2.9–7.5; *p* < 0.001) for DeepSeek vs. Oncologist.

Post hoc Dunn tests with Holm adjustment demonstrated that, for ChatGPT vs. Oncologist and DeepSeek vs. Oncologist, concordance grades in Stage I differed significantly from those in Stages II–IV, whereas no significant difference was observed between Stages II and III. For BC, significant differences were observed between Stage I and all subsequent stages, as well as between Stage II and Stages III and IV.

### 3.4 Variability and dispersion analysis

To assess the concordance of treatment recommendations, descriptive statistics were computed for each cohort stratified by disease stage. Across all groups, ChatGPT versus DeepSeek comparisons demonstrated lower dispersion relative to AI-to-oncologist comparisons, suggesting greater inter-AI agreement than AI-to-clinician agreement (Table 3).

**Table 3:** Treatment recommendation concordance score variability.

Cohort/Comparison	Mean	SD	CV (%)
Overall (*N* = 213)			
ChatGPT vs. DeepSeek	77.42	18.82	24.31%
ChatGPT vs. Oncologist	38.74	22.45	57.95%
DeepSeek vs. Oncologist	40.92	21.18	51.76%
Stage I (*N* = 24)			
ChatGPT vs. DeepSeek	82.13	12.45	15.16%
ChatGPT vs. Oncologist	41.52	19.33	46.56%
DeepSeek vs. Oncologist	44.83	17.65	39.37%
Stage II (*N* = 75)			
ChatGPT vs. DeepSeek	76.51	18.12	23.68%
ChatGPT vs. Oncologist	40.21	21.05	52.35%
DeepSeek vs. Oncologist	42.15	20.14	47.78%
Stage III (*N* = 70)			
ChatGPT vs. DeepSeek	77.24	20.35	26.35%
ChatGPT vs. Oncologist	35.84	24.11	67.27%
DeepSeek vs. Oncologist	38.62	22.56	58.42%
Stage IV (*N* = 44)			
ChatGPT vs. DeepSeek	73.58	21.45	29.15%
ChatGPT vs. Oncologist	37.12	23.88	64.33%
DeepSeek vs. Oncologist	38.2	22.12	57.91%

Analysis of the dispersion in treatment recommendation scores across groups revealed significant heterogeneity in score variances among different disease stages (Across Stages), as indicated by the Levene test (W = 4.82, *p* = 0.009). Given the potential non-normal distribution characteristics of medical clinical decision-making data, a median-based Brown–Forsythe robust test for equality of variances was further employed for verification. The test results confirmed that the differences in variances among the groups were statistically significant (W = 5.14, *p* = 0.006), indicating that the level of consistency in treatment recommendations exhibited significant fluctuations across different clinical contexts.

## 4 Discussion

This study reveals a structured pattern of divergence between LLM and oncologists that extends beyond simple differences in mean accuracy. Rather than demonstrating isolated performance gaps, our findings indicate the presence of a dual-layer phenomenon characterized by (1) systematic accuracy hierarchy and (2) structurally distinct variability patterns across clinical complexity gradients.

In this cohort of 213 breast cancer cases, large language models (LLMs) demonstrated higher evaluation scores than human oncologists. DeepSeek achieved the highest mean score with the lowest variance, followed by ChatGPT, whereas oncologists exhibited lower and more dispersed ratings. Between-group differences were statistically significant, and pairwise comparisons consistently confirmed a stable ranking of DeepSeek > ChatGPT > Oncologist, suggesting that these disparities reflect structural differences in decision-making logic rather than random variation. The small standard deviation observed for DeepSeek further indicates high internal consistency across cases.

Both DeepSeek and ChatGPT demonstrated high accuracy and internal consistency in breast cancer diagnostic and therapeutic tasks, consistent with prior studies, with DeepSeek showing superior performance in certain oncologic scenarios (5, 15). Their treatment recommendations aligned with Chinese and NCCN guidelines, and AI systems—particularly DeepSeek—provided more comprehensive options than oncologists, attributed to its mixture-of-experts architecture and extensive Chinese medical training (16, 17). The expert observed that both DeepSeek and ChatGPT provide more comprehensive treatment options than Oncologist, and DeepSeek also offer more option than ChatGPT.

The observed quantitative patterns can be explained by how LLMs generate recommendations. Both DeepSeek and ChatGPT rely on probabilistic pattern matching grounded in large-scale training data, clinical guidelines, and consensus documents (18). DeepSeek's combination of higher mean scores and lower variance suggests more uniform alignment with standardized treatment logic across cases. In contrast (19), ChatGPT displayed greater score variability, consistent with its sensitivity to linguistic and contextual variations in case descriptions, leading to more fluctuating evaluations (20).

By comparison, the lower and more dispersed scores assigned by oncologists likely reflect the incorporation of real-world clinical considerations that are not fully captured in structured case summaries. These include patient preferences, anticipated treatment tolerance, comorbidities, and prognostic uncertainty. Importantly, clinicians' decisions are also constrained by several structural factors that are typically invisible to guideline-based evaluation frameworks, including drug accessibility at the time of treatment, medical insurance reimbursement policies, patients' economic capacity and preferences, and the historical state of available evidence (21, 22). Consequently, clinicians' choices may deviate from retrospectively “optimal” recommendations derived from current guidelines, yet remain entirely rational within their original clinical context. Thus, AI–human discrepancies should be interpreted not merely as performance gaps, but as reflections of fundamentally different decision-making frameworks (23, 24). The critical insight lies in variance compression: LLM outputs clustered tightly around guideline-concordant solutions, forming an algorithmic consensus structure, whereas oncologist recommendations showed broader distribution across clinically acceptable options therefore, the observed difference between AI and human performance should not be simplistically attributed to a performance gap. Instead, it untangles a fundamental structural divide between a probabilistic knowledge compression model and a contextualized experiential integration framework. This divide leads to convergence in standardized scenarios but results in inevitable and justifiable divergence in high-uncertainty, complex scenarios (25–27).

The high concordance observed between AI systems and clinicians in early-stage (I–III) breast cancer further supports this interpretation. In this study, both DeepSeek and ChatGPT demonstrated high consistency rates (60%–95%) in treatment recommendations for stages I–III, with stage I exceeding 95%. This aligns with existing literature showing that authoritative guidelines such as NCCN, ESMO, and CSCO have established clear and stable adjuvant treatment pathways for early breast cancer, supported by high-level evidence and minimal regional variation (28, 29). Given that LLM training corpora contain large volumes of standardized guidelines and systematic reviews, AI systems naturally generate highly consistent recommendations in evidence-rich, protocol-driven scenarios (12).

In contrast, markedly greater divergence in treatment recommendations was observed across all groups in advanced (stage IV) cases. This variability reflects the intrinsic complexity of late-stage breast cancer management rather than arbitrary differences in option selection. Stage IV disease is associated with pronounced biological heterogeneity, including multiple organ metastases and dynamically evolving biomarkers such as HER2 and PD-L1, leading to diverse disease trajectories progression (30, 31). In such contexts, clinical guidelines often present multiple parallel options, with emerging and legacy evidence coexisting and without a clearly defined optimal pathway.

Traditional evaluation frameworks assume expert consensus as ground truth. However, the present data demonstrate that clinical practice itself exhibits substantial internal heterogeneity (32, 33). Thus, AI systems may not be deviating from a unified gold standard, but rather from a heterogeneous clinical landscape shaped by contextual and temporal realities. This observation suggests that future evaluation frameworks should incorporate structured agreement and dispersion metrics alongside accuracy-based measures.

Moreover, the gap between “guideline ideals” and “clinical reality” becomes particularly prominent in advanced disease. Access to novel therapies—such as antibody–drug conjugates or PARP inhibitors—is heavily influenced by healthcare system capacity, insurance coverage, and patients' economic burden (34). As a result, clinicians may prioritize treatments that balance feasibility, toxicity, and affordability (35), whereas AI-generated recommendations tend to reflect the most up-to-date evidence-based options without accounting for real-world constraints. Additionally, advanced cancer treatment often spans many years. Real-world clinician decisions reflect the historical availability of drugs, diagnostic tools, and prevailing medical knowledge at the time, whereas AI systems generate optimal recommendations based on the latest consolidated evidence, further widening apparent AI–human discrepancies.

This study also underscores a current limitation of AI-assisted systems: informational latency. LLMs primarily rely on published literature and established guidelines and may not immediately incorporate cutting-edge findings presented at major oncology conferences or therapies still under clinical investigation (36). Consequently, AI systems function as highly structured but partially static knowledge repositories and cannot replace clinicians' ongoing engagement with rapidly evolving evidence.

Finally, cancer care is inherently individualized. Not all guideline-recommended or AI-suggested treatments are appropriate for every patient. Clinicians may intentionally deviate from guideline-preferred options due to patient-specific factors, including comorbidities, treatment tolerance, personal values, and social circumstances (37). Apparent discrepancies between AI-generated and clinician-selected treatments therefore often reflect the complexity of real-world decision-making rather than differences in clinical competence.

In summary, the high concordance between AI systems and clinicians in early-stage breast cancer highlights the strong guideline dependence of evidence-rich clinical scenarios and suggests that AI can generate reliable, expert-like treatment plans when pathways are clear. Conversely, divergences observed in advanced disease underscore the limitations of guideline-driven AI systems and reaffirm the indispensable role of human clinical judgment in navigating uncertainty, contextual constraints, and individualized patient care.

### 4.1 Limitation

This study has several limitations. First, our evaluation focused primarily on accuracy and concordance metrics, without assessing other clinically relevant dimensions such as explainability, fabricated references, or unsafe recommendations. While we acknowledge that general-purpose LLMs inherently carry a theoretical risk of generating non-evidence-based outputs—particularly in complex scenarios—we did not observe frequent factual errors in this cohort. Second, the number of participating oncologists was relatively limited, which may restrict the generalizability of the findings. Future studies should include larger and more diverse cohorts of clinicians across multiple institutions to improve external validity. Third, the large language models evaluated in this study were originally designed for general-purpose use rather than specifically trained for medical professionals, which may increase the risk of hallucinated or non-evidence-based outputs in complex clinical scenarios. It is important to note that the LLMs in this study operated under “idealized” conditions, as inputs were limited to clinical pathology and did not include real-world constraints such as drug accessibility, insurance coverage, or patient preference. Consequently, the superior performance of LLMs reflects guideline concordance​ rather than real-world feasibility.

## 5 Conclusion

DeepSeek and ChatGPT demonstrate remarkable proficiency in generating standardized, guideline-aligned breast cancer treatment plans, with DeepSeek showing superior consistency in the Chinese clinical context. While LLMs excel in protocol-driven early-stage cases, their “information lag” and inability to account for real-world patient complexity (e.g., economic factors, toxicity tolerance) remain limitations. LLMs currently serve as powerful decision-support tools for standardizing oncology care rather than replacing the individualized judgment of specialists.

## Data Availability

The raw data supporting the conclusions of this article will be made available by the authors, without undue reservation.
